# Laparoscopic cystoprostatectomy for bladder cancer in a male patient combined with open ileal conduit urinary diversion

**DOI:** 10.1590/S1677-5538.IBJU.2014.0632

**Published:** 2017

**Authors:** Rafael P. Arruda, Mirandolino B. Mariano, Clovis Fraga T. Pereira, Guilherme C. Lima, Thiago N. Lessa, Moacir C. de A.

**Affiliations:** 1 Departamento de Urologia, Instituto de Medicina Integral Prof. Fernando Figueira - IMIP, Recife - PE, Brasil

## BACKGROUND AND OBJECTIVES

Although open radical cystectomy remains the gold standard treatment for muscle invasive bladder cancer (MIBC), laparoscopic cystoprostatectomy (LCP) has proven to be safe, and along with the robot-assisted technique is gaining more space. In this video, we describe the steps of LCP, featuring our approach. With the combination of extirpative stage surgery with conventional reconstructive part (Bricker ileal conduit), our goal is to offer the advantages of a minimally invasive approach with oncological and perioperative safety.

## MATERIALS AND METHODS

Patient, 57 years ago, T2aN0M0, with erectile dysfunction and ECOG-0. Neoadjuvant chemotherapy with gemcitabine and cisplatin was used (4 cycles). The bowel was prepared by oral self-administration of 1 liter of electrolyte solution. Prophylaxis with a cephalosporin was administrated for 5 days and Enoxaparin 40mg was administered preoperatively and until postoperative day 8. We used the five-port transperitoneal approach. The surgery was performed in May/2014 at the Institute of Integrative Medicine Prof. Fernando Figueira - IMIP, Recife-PE/Brazil.

## RESULTS

The operative time was 160 minutes. The oral diet was resumed on the 3 ^rd^ postoperative day (POD). Estimated blood loss of 700ml. Postoperatively, recovery was uneventful and the patient was discharged on the 4 ^th^ POD. During the 10 month-follow-up, no major complications occurred. Only a left hydronephrosis (G1) was found in abdominal US performed in the 3 ^rd^ month of follow-up, but didn’t show complications so far. There wasn’t tumor recurrence and no adjuvant therapy was required.

## CONCLUSIONS

LCP is a feasible and safe procedure, with a promising future in minimally invasive surgical procedures technique.

## ARTICLE INFO

Available at: http://www.intbrazjurol.com.br/video-section/arruda_169_170/

Int Braz J Urol. 2017; 43 (Video #2): 169-70
